# Anaplastic Transformation of Papillary Thyroid Cancer in the Retroperitoneum

**DOI:** 10.1155/2015/241308

**Published:** 2015-08-16

**Authors:** James P. Solomon, Fang Wen, Lily J. Jih

**Affiliations:** ^1^Department of Pathology, University of California, San Diego, 9500 Gilman Drive, La Jolla, CA 92093, USA; ^2^Department of Pathology, Veteran Administration Medical Center, La Jolla, 3350 La Jolla Village Drive (113), San Diego, CA 92161, USA

## Abstract

Anaplastic thyroid carcinoma is an aggressive variant of thyroid cancer that in most cases arises from anaplastic transformation of terminally differentiated thyroid carcinomas. This process usually occurs in the thyroid or cervical lymph nodes. Anaplastic transformation in distant metastatic sites is exceedingly rare, only previously documented in a few case reports. We report a rare case of anaplastic transformation of papillary thyroid carcinoma within a large retroperitoneal metastasis in a 64-year-old male 30 years after the initial diagnosis.

## 1. Introduction

Anaplastic thyroid carcinoma (ATC) is a rare form of thyroid carcinoma that is associated with an extremely poor prognosis. Most ATCs arise in the thyroid itself or in cervical lymph node metastases from anaplastic transformation of preexisting terminally differentiated thyroid carcinomas such as papillary or follicular thyroid carcinoma [1]. While terminally differentiated thyroid cancer can metastasize to distant sites, anaplastic transformation occurring within a distant metastatic site is a very rare phenomenon, with only a few reported cases in the literature [2–8]. Here, we report a rare case of anaplastic transformation of papillary thyroid carcinoma (PTC) in a retroperitoneal metastasis in a patient who underwent total thyroidectomy almost 30 years earlier.

## 2. Case Presentation

**Figure 1:**
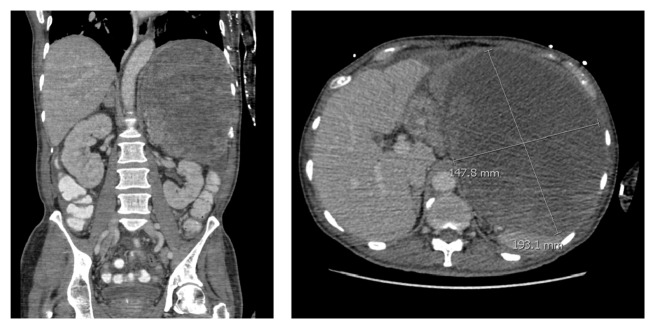
Abdominal CT scans demonstrating a 20 cm mass in the left upper quadrant retroperitoneum.

A 64-year-old male presented with persistent abdominal pain, and on imaging, he was found to have a 20 cm abdominal mass causing mass effect on the stomach, pancreas, left adrenal gland, and kidney (Figure 1). The patient had a history of PTC that was initially discovered in 1985, and at that time, he underwent total thyroidectomy and had postoperative radioactive iodine treatment. In 1999, he was found to have metastatic disease to the cervical lymph nodes and underwent a second round of radioactive iodine therapy. In 2005, he developed metastatic disease to the left axilla and was treated with local resection. In 2013, the patient presented with abdominal pain and was found to have a large retroperitoneal tumor that demonstrated strong FDG uptake on PET-CT, but radioactive iodine testing was not performed. Fine needle aspiration of the retroperitoneal mass performed at an outside institution showed a papillary neoplasm that was positive for TTF-1 and thyroglobulin, consistent with metastatic PTC. The patient was referred to our institution six months later for palliative resection of the tumor. His postoperative course was complicated by large bowel obstruction and sepsis. The patient expired 3 weeks after surgery.

**Figure 2:**
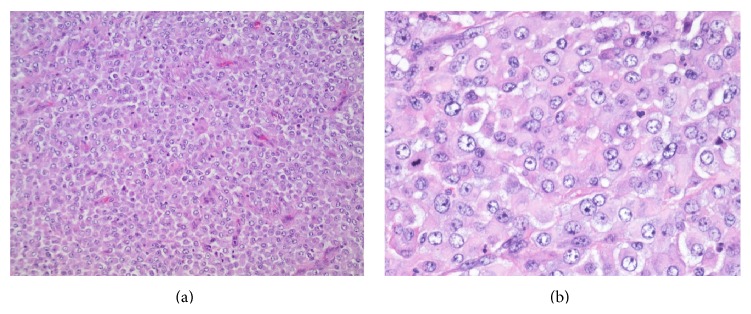
Microscopic examination of the retroperitoneal mass. There were sheets of undifferentiated cells with no papillary architecture seen ((a) 100x). The malignant cells had rhabdoid features with abundant granular eosinophilic cytoplasm, eccentric, vesicular nuclei, and prominent nucleoli. Mitoses were readily identified ((b) 400x).

**Figure 3:**
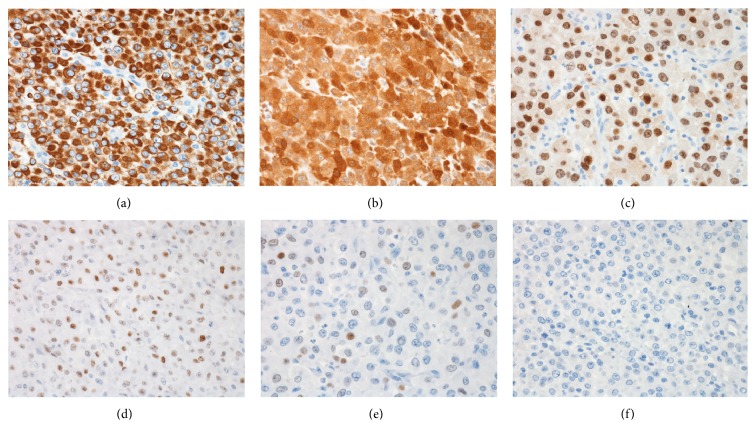
Immunohistochemical features of the tumor cells. The tumor cells showed diffuse positivity for AE1/AE3 (a), galectin-3 (b), and PAX-8 (c). There was patchy positivity for p53 (d). The tumor cells showed only weak focal positivity for TTF-1 (e) and were negative for thyroglobulin (f). All photographs at 400x magnification.

*Histological Findings*. Grossly, the resected retroperitoneal mass measured 37 cm in greatest dimension and it was mostly necrotic with minimal viable areas. Extensive sampling showed sheets of pleomorphic cells with rhabdoid features including abundant granular eosinophilic cytoplasm and eccentric, vesicular nuclei with prominent nucleoli. Mitoses were readily identified (Figure 2). Due to the unusual morphology and location, immunohistochemical stains were performed. The tumor cells were strongly positive for AE1/AE3, galectin-3, PAX8 (Figures 3(a)–3(c)), and vimentin, with patchy positivity for p53 (Figure 3(d)). There was focal weak staining for TTF-1 (Figure 3(e)), CK7, CK19, and S100. Thyroglobulin (Figure 3(f)), HBME-1, CK20, OCT3/4, CD117, CD45, and HMB45 were negative (Ventana Medical Systems, Tucson, AZ). Given the patient's long standing history of recurrent and metastatic PTC and the overall immunohistochemical profile, the findings were consistent with anaplastic transformation in a metastatic PTC.

## 3. Discussion

The retroperitoneal mass showed an undifferentiated tumor composed of sheets of malignant cells with eccentric nuclei, prominent nucleoli, and abundant eosinophilic cytoplasm. Although prior fine needle aspiration from an outside institution revealed tumor cells with papillary architecture consistent with metastatic PTC, no PTC component was identified in the resected specimen despite extensive sampling. Given the history of recurrent and metastatic PTC, anaplastic transformation of PTC was considered at the top of the differential diagnosis. However, because of the unusual location and the lack of identifiable PTC component in the resected retroperitoneal mass, other tumors were also considered. Differential diagnosis at this site included high-grade sarcoma, metastasis from other unknown primary, high-grade lymphoma, and melanoma. Additional immunohistochemistry was therefore performed for further characterization. The presence of strong and diffuse positivity for AE1/AE3 confirmed carcinoma. The tumor cells were negative for thyroglobulin and showed only focal staining for TTF-1, which often occurs in anaplastic transformation, as studies have demonstrated loss of thyroglobulin and TTF-1 expression in ATC [9]. The tumor cells were also positive for PAX-8, which is a transcription factor expressed in both normal and neoplastic thyroid, kidney, and female genital tract [10]. PAX-8 has been reported to be a reliable marker of thyroid origin, even after anaplastic transformation [11]. The tumor cells also expressed galectin-3 and focally expressed CK19, additional markers that have been shown to be positive in PTC [12]. Positivity for p53, which is correlated with mutations in the gene that expresses the protein, is also often seen in a majority of ATC [6]. The overall immunohistochemical profile supported the diagnosis of anaplastic transformation of PTC.

ATC is the most aggressive type of thyroid neoplasm, with a 5-year survival rate of less than 10% and a mean survival time of six months after diagnosis [13]. Most patients present with rapidly enlarging neck mass and at least 40% of the patients have distant metastases at the time of diagnosis. Poor prognostic factors include higher age at diagnosis, male gender, leukocytosis, tumor size, extrathyroidal invasion, and presence of distant metastases [14]. While surgical treatment and radiotherapy have been shown to improve survival, the prognosis remains dismal [14].

Most cases of ATC are thought to arise from preexisting differentiated thyroid carcinoma. Some cases may develop de novo from preexisting multinodular goiter. Anaplastic transformation typically occurs within the thyroid gland and the cervical lymph nodes, and foci of ATC can occasionally be seen in these locations at the time of initial surgical management for differentiated thyroid carcinoma [1, 2]. Autopsy studies have demonstrated that ATC is often widely metastatic at the time of death, with two or more metastatic sites found in the vast majority of cases. The metastatic sites include the lungs, intrathoracic lymph nodes, cervical lymph nodes, pleura, adrenal glands, liver, brain, heart, and retroperitoneal lymph nodes [1].

Anaplastic transformation of a differentiated thyroid carcinoma at the distant site is exceedingly rare, only previously documented in a few case reports [2–8]. Only one of these reports described anaplastic transformation in a retroperitoneal metastasis [5], with other reported sites including the liver [6], pelvis [8], soft tissue of the shoulder [4], submandibular gland [7], and the lungs [2, 3]. The anaplastic transformation at the metastatic site can occur many years after the initial diagnosis and surgical treatment for the initial differentiated PTC; in these reported cases, the time from initial thyroidectomy to identification of anaplastic thyroid carcinoma at the metastatic site ranged from 7 to 17 years [2–8]. In many of these cases, the patients often have widespread metastatic PTC, and anaplastic transformation in distant metastases was identified on autopsy studies.

In the current case, the patient had a known history of metastatic PTC to cervical and axillary lymph nodes before presenting with a large retroperitoneal mass 30 years later. The retroperitoneal mass had evidence of PTC by cytological examination, suggesting that anaplastic transformation had occurred in the retroperitoneal mass. In addition, it has been proposed that postoperative radioactive iodine therapy can contribute to anaplastic transformation of PTC [6, 15], and in this case, the patient had two separate rounds of radioactive iodine therapy approximately 15 years apart.

In conclusion, this case demonstrates the rare occurrence of anaplastic transformation of PTC in an unusual distant site 30 years after the initial presentation. To our knowledge, this is the second reported case of anaplastic transformation of PTC within a retroperitoneal metastasis. As ATC has a much poorer prognosis than other types of thyroid carcinoma, its presence should always be reported. While it is known that thorough sampling and examination of thyroidectomy specimens and regional lymph node dissections should be performed, this case emphasizes that anaplastic transformation can occur even in distant metastases. It is therefore recommended that all resection specimens in patients with a history of thyroid carcinoma, including distant metastases, should be thoroughly sampled for the presence of ATC.
